# Transgender Men and Transmasculine One-on-One and Group-Delivered Empowerment for Targeted HIV Reduction (TOGETHR) Study: Protocol for a Digital Factorial Randomized Controlled Trial

**DOI:** 10.2196/76831

**Published:** 2025-10-20

**Authors:** Sari L Reisner, Ayden I Scheim, S Wilson Cole, Andrea L Wirtz, Tonia Poteat, Matthew J Mimiaga, Mark A Marzinke, Alek I Meyer, Kevin Smith, David R Pletta, Kenneth H Mayer

**Affiliations:** 1 Department of Epidemiology School of Public Health University of Michigan Ann Arbor United States; 2 The Fenway Institute Fenway Health Boston, MA United States; 3 Drexel University Philadelphia United States; 4 Department of Health, Behavior, and Society, School of Public Health Johns Hopkins University Baltimore United States; 5 Queery Research Consulting Glen Burnie, MD United States; 6 Department of Epidemiology School of Public Health Johns Hopkins University Baltimore United States; 7 Division of Healthcare in Adult Populations School of Nursing Duke University Durham United States; 8 Department of Epidemiology School of Public Health University of California, Los Angeles Los Angeles United States; 9 Department of Pathology School of Medicine Johns Hopkins Medicine Baltimore United States; 10 Department of Medicine Medical School Harvard University Boston United States; 11 Department of Medicine Beth Israel Deaconess Medical Center Boston United States

**Keywords:** HIV prevention, transgender, preexposure prophylaxis

## Abstract

**Background:**

In the United States, transgender men and transmasculine people who have sex with men (TMSM) face an increased risk of HIV and encounter unique barriers to HIV prevention services, including preexposure prophylaxis (PrEP). Peer-based, digitally delivered support interventions that address these barriers in 1-on-1 or small-group settings may be effective in increasing PrEP engagement.

**Objective:**

This study aims to compare the efficacy of digitally delivered individual and small-group peer-based strategies for improving PrEP uptake and adherence among at-risk adult TMSM without HIV.

**Methods:**

The Transmasculine One-on-One and Group Empowerment for Targeted HIV Reduction (TOGETHR) study was a digital, open-label, randomized 2×2 factorial trial (1:1:1:1 randomization) of peer-delivered HIV prevention strategies designed to increase PrEP engagement in 300 PrEP-indicated TMSM without HIV. Participants were randomized to 1 of 4 conditions: (A) standard of care (SOC) package (information and PrEP locator tools), (B) SOC + PrEP for TMSM (PrEP4T; individual peer-based HIV prevention intervention), (C) SOC + LifeSkills for TMSM (LS4TM; group peer-based HIV prevention intervention), or (D) SOC + PrEP4T and LS4TM (both individual and group interventions). Interventions were delivered over a 6-week period. The study enrolled adults 18 years or older who were “transgender men or transmasculine,” sexually active with 1 or more partners who had a penis and were assigned male sex at birth, living without HIV, behaviorally at risk for HIV acquisition, and residing in geographic hot spots identified in the US Ending the HIV Epidemic initiative.

**Results:**

Study protocols and procedures were codeveloped with our community advisory board, including eligibility criteria, operationalization of geographic stratification, study branding, and detailed recruitment plans. Recruitment and enrollment began on April 1, 2024, and as of March 20, 2025, 103 TMSM had been enrolled. The median age was 26 (IQR 24-31) years. Among the 103 participants, 39 (37.9%) identified as Black, Indigenous, and People of Color, and 18 (17.5%) as Latine; 36 (35.0%) identified as “transgender man or trans man”; 31 (30.1%) as transmasculine; and 15 (14.6%) as nonbinary, genderqueer, or gender nonconforming. Additionally, 69 (67.0%) had at least a four-year college degree, and only 7 (6.8%) had no health insurance. Granger causality tests indicated that recruitment activities via social media (P=.006) and transgender-inclusive dating platforms (P=.02) were most effective and predicted higher baseline enrollment survey completion 2 weeks later. On March 21, 2025, the National Institutes of Health terminated the grant award, citing gender ideology and stating, “it is the policy of NIH not to prioritize such research programs.”

**Conclusions:**

This study was part of a critical research pathway to identify effective strategies for preventing HIV acquisition in TMSM, a group traditionally underserved in HIV prevention, and to inform prevention packages that incorporate peer-delivered interventions. Strong engagement with the TMSM community was central to the study’s success and provided valuable insights for future HIV prevention research and practice.

**Trial Registration:**

ClinicalTrials.gov NCT06182280; https://clinicaltrials.gov/ct2/show/NCT06182280

**International Registered Report Identifier (IRRID):**

DERR1-10.2196/76831

## Introduction

### HIV Epidemiology and Acquisition Risk

Transgender men and transmasculine people—those who identify as men or masculine and were assigned female at birth—have not been centered in HIV prevention efforts until recently [1-3]. Transgender people, who comprise an estimated 1.4 million US adults, are identified as a key population in the 2021-2025 US National Strategic Plan to end the HIV epidemic [4,5]. In a 2019 meta-analysis, HIV prevalence among transgender men in the United States was 3.2%—10 times greater than in the general US population [6]. Many transgender men and transmasculine people are men who have sex with men (TMSM) and engage in sexual activity with cisgender (cis) males and with partners of other genders who have a penis [7]. TMSM, especially those who engage in condomless genital or anal sex or who share needles for illicit drug use and hormones, are at risk for HIV and require tailored HIV prevention strategies [8-11]. However, no efficacious HIV prevention interventions currently exist for this population [1]. Research is needed to develop interventions that facilitate access to biobehavioral HIV prevention for at-risk TMSM, particularly those in geographic hot spots with a high HIV epidemic burden [4].

### TMSM Barriers to HIV Prevention

TMSM require HIV prevention services that address their unique situated vulnerabilities and gender affirmation needs [12]. Most TMSM have not undergone genital surgery and may engage in receptive genital and anal sex, which often goes unreported to sexual health providers [8,13]. Gender nonaffirmation from cis male sexual partners is common and is associated with behavioral risk for HIV acquisition in TMSM [14]. TMSM also express concerns about concomitant preexposure prophylaxis (PrEP) and testosterone use for medical gender affirmation, which may reduce interest in and uptake of PrEP among TMSM with HIV behavioral risk [15]. Finally, TMSM are at increased risk of mental health morbidity (eg, depression, substance use), which is associated with HIV risk [7]. However, TMSM face barriers to accessing HIV prevention services, and tailored services remain lacking [15]. These barriers include discrimination that leads to care avoidance, misconceptions that TMSM are not at risk for HIV, and logistical challenges such as scheduling medical appointments [14-16]. The noninclusion of TMSM in PrEP trials and promotion further deters their use of PrEP [1]. Thus, effective HIV prevention for TMSM will need to remove barriers to care and address gender affirmation needs, while also building awareness, motivation, and behavioral skills necessary to prevent HIV among all transgender and MSM populations [17-19].

### Peer-to-Peer Individual- and Group-Based Strategies for HIV Prevention

Evidence-based peer strategies that empower communities through social support, stigma reduction, and skills building are widely accepted in transgender communities [20-23]. Peer navigation interventions are individualized, using 1-on-1 sessions led by trusted peers with shared lived experiences. Peer navigation has demonstrated success in improving outcomes among people with HIV [24-32], including transgender women [33-35]. It has also been effective in increasing PrEP uptake and adherence [36-38], and shows promise for transgender women [37,39]. While peer navigation is endorsed by TMSM [17], no interventional efficacy research has yet been conducted.

Our team has previously adapted and implemented peer navigation interventions for transgender communities to increase linkages to health care services. This includes the Gender-Affirming Clinical and Public Health Model at Fenway Health (Fenway), which employs individualized 1-on-1 peer health navigation to support care engagement [32,40]. Peer health navigation typically involves 4 structured sessions over 6 months of follow-up, and evaluation of Fenway’s program has demonstrated high feasibility and acceptability, with increased health care engagement outcomes within 60 days [41]. One-on-one sessions incorporate goal setting, skills building, assessment, planning, and navigation. Peer navigation has also been shown to increase linkage to medical, behavioral, and HIV prevention services (eg, PrEP) [41,42]. The Fenway peer navigation model has been disseminated nationally [43] and digitally [44]. Our team further replicated peer navigation with transgender people in Peru for HIV prevention [45], highlighting its adaptability, utility, and broad transferability.

Another evidence-based HIV prevention strategy used in transgender communities is group-based peer interventions, which involve peer-facilitated group sessions [20,21,23,46]. A pilot study of LifeSkills for Men (LS4M) [20], a small group–based behavioral intervention designed to reduce HIV risk and the mental health effects of stigma in TMSM, found high acceptability and positive trends in health outcomes. LS4M was adapted from LifeSkills [21], an efficacious intervention for transgender women. The LS4M curriculum consisted of 8 hours of content delivered across 4 weekly sessions. The pilot demonstrated that LS4M was feasible, highly acceptable, and associated with promising improvements over 4 months of follow-up, including increased transgender support and reductions in mental health severity, sexual risk, and gender nonaffirmation [20].

Evidence supports peer-delivered interventions for HIV prevention; however, few studies have compared individual and group formats or evaluated combined delivery [47,48]. Research is needed to assess the individual and combined effects of 1-on-1 and group-based peer-delivered strategies. Individualized interventions are tailored to the specific needs of TMSM, while group-based interventions affirm TMSM not only as individuals but also collectively as members of a community.

### Conceptual Frameworks

**Figure 1:**
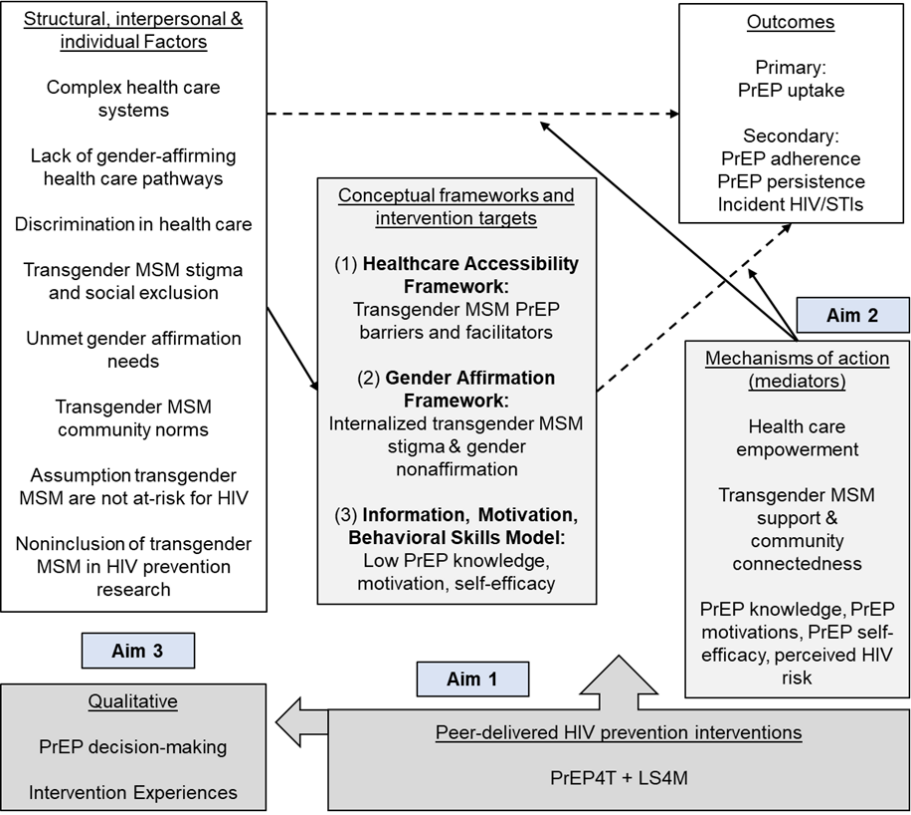
Conceptual frameworks mapped to study aims. LS4TM: LifeSkills for Transgender Men and Transmasculine People; MSM: men who have sex with men; PrEP: preexposure prophylaxis; PrEP4T: PrEP for transgender men and transmasculine people; STI: sexually transmitted infection.

Three conceptual frameworks informed the study (Figure 1). First, the Healthcare Accessibility Framework [49] highlights systemic and population-specific barriers to HIV prevention for TMSM, such as avoidance of care due to stigma, misconceptions that transgender men are not at risk for HIV, perceived difficulty navigating PrEP logistics [14-16], and the need for additional services (eg, mental health). It also emphasizes facilitators of access, such as health care empowerment [50]. Second, the Gender Affirmation Framework [7,51] underscores that being affirmed in one’s gender is a key health determinant and that gender affirmation is not “one-size-fits-all.” Many TMSM do not have or want genital surgery and are therefore at increased risk of HIV when engaging in receptive anal and vaginal sex [8,13]. Stigma and gender nonaffirmation also negatively affect HIV risk-taking and PrEP decision-making [14,52]. Gender-affirming HIV prevention that addresses TMSM-specific vulnerabilities is essential. Third, the Information, Motivation, and Behavioral Skills (IMB) model [53] is an evidence-based behavior change framework that targets increasing information (eg, PrEP knowledge), motivation (eg, attitudes, perceived benefits/risks), and behavioral skills (eg, self-efficacy) for TMSM. Effective HIV prevention for TMSM must integrate health care access, gender affirmation, and IMB constructs [17-19].

### Study Objective and Aims

The goal of the Transmasculine One-on-One and Group Empowerment for Targeted HIV Reduction (TOGETHR) study was to evaluate the effectiveness of digitally delivered individual and small-group peer-based strategies in improving PrEP uptake and adherence among at-risk, HIV-negative adult TMSM.

Applying the Healthcare Access [49], Gender Affirmation [7,51], and IMB [53] frameworks, we conducted a digital, open-label, randomized 2×2 factorial trial (1:1:1:1 allocation) of peer-delivered HIV prevention strategies to increase PrEP uptake among TMSM (n=300) residing in Ending the HIV Epidemic (EHE)–prioritized geographic hot spots in the United States. The specific aims were to:

- Aim 1. Compare the efficacy of 4 peer-delivered HIV prevention strategies implemented over 6 weeks to increase PrEP uptake (primary outcome) among 300 PrEP-indicated, HIV-negative TMSM, which include
  (A) standard of care (SOC)
  digital PrEP locator and a library of syndicated HIV prevention, sexual health, and antistigma materials;
  (B) SOC + PrEP for transgender men and transmasculine people (PrEP4T; 1-on-1 PrEP peer navigation); (C) SOC + LifeSkills for Transgender Men and Transmasculine People (LS4TM; peer-delivered group-based PrEP behavioral intervention); or (D) SOC + PrEP4T + LS4TM.
- Hypothesis 1.1:
  Rates of PrEP uptake will be higher in (B) SOC + PrEP4T and (C) SOC + LS4TM conditions compared with (A) SOC (main effects).
- Hypothesis 1.2:
  PrEP uptake rates will be highest for participants randomized to (D) SOC + PrEP4T + LS4TM compared with (A) SOC (additive effects).
- Aim 2. Examine the mechanisms through which PrEP4T and LS4TM impact PrEP uptake using causal mediation analysis.
- Hypothesis 2.1:
  Health care access empowerment, transgender community support for gender affirmation, and IMB constructs will mediate the relationship between PrEP4T, LS4TM, and PrEP uptake.
- Aim 3. Explore PrEP decision-making processes and intervention experiences through in-depth interviews (IDIs; n=40; 10 per condition) to inform future scale-up and implementation of these interventions.

## Methods

### Study Design

The TOGETHR study was a collaboration between the University of Michigan and The Fenway Institute at Fenway Health. The Fenway Institute led study management, including recruitment and retention, enrollment, intervention implementation, and data collection. The University of Michigan was responsible for data analysis. Investigators from Johns Hopkins University, Duke University, Drexel University, and the University of California, Los Angeles also contributed to the study.

TOGETHR was a digitally delivered, open-label, randomized 2×2 factorial trial of peer-delivered HIV prevention strategies. The design included 2 factors: individualized 1-on-1 peer navigation (PrEP4T vs none) and group-based behavioral intervention (LS4TM vs none). The primary outcome was PrEP uptake, confirmed biologically. Secondary outcomes were PrEP adherence, defined as tenofovir diphosphate (TFV-DP) concentrations >700 fmol/punch (corresponding to >4 doses per week) [54], and PrEP persistence, defined as 2 consecutive dried blood spot (DBS) measures indicating PrEP use. Participants were randomized 1:1:1:1 to the 4 study conditions, with stratification by ethnoracial identity (Black, Indigenous, and People of Color [BIPOC] vs White non-Latine) and by Movement Advancement Project (MAP) state lesbian, gay, bisexual, transgender, and queer (LGBTQ) equality policy tally (“high, medium, or fair” vs “low or negative”) [55]. The enrollment target was approximately 50% BIPOC participants. The study protocol was developed in accordance with the 2025 Standard Protocol Items: Recommendations for Interventional Trials (SPIRIT) guidelines to ensure methodological rigor, transparency, and completeness in trial reporting [56].

TOGETHR was funded by the National Institutes of Health (NIH; R01MH129175). On March 21, 2025, however, the NIH prematurely terminated the grant, citing its focus on “gender identity.” The justification given was that, under the Trump administration (commencing January 20, 2025), “it is the policy of NIH not to prioritize such research programs." In April 20205, the American Civil Liberties Union (ACLU), Protect Democracy, and the Center for Science in the Public Interest filed a lawsuit on behalf of researchers, the American Public Health Association (APHA), and others challenging the abrupt termination of research grants on disfavored topics and populations by NIH. The current grant was included in the lawsuit. The grant was re-instated on July 9, 2025; however, litigation is ongoing and the future of the project remains uncertain [57].

### Study Population: Eligibility Criteria

Inclusion criteria were (1) age ≥18 years; (2) assigned female sex at birth and identified as a “man, transgender man, or transmasculine”; (3) had sex in the past 3 months with a partner who had a penis and was assigned male sex at birth; (4) access to a smartphone or computer with internet; (5) HIV-negative at baseline (confirmed); (6) reported behavioral HIV risk in the past 3 months, including at least one of the following: (i) receptive vaginal/frontal or anal sex with an assigned male sex at birth partner with a penis; (ii) sharing needles or syringes for illicit drug use or hormones; (iii) self-reported sexually transmitted infection diagnosis; (7) residence in a US EHE-targeted geographic hot spot (48 counties, Washington, DC, San Juan, Puerto Rico—where >50% of HIV diagnoses occurred in 2016-2017—or 7 states with substantial rural HIV diagnoses; see Table S1 in Multimedia Appendix 1); (8) willing and able to provide informed consent in English; and (9) comfortable reading and conversing in English.

### Community Participation: A Participatory Population Perspective

TOGETHR employed a participatory population approach, working with—rather than on—the TMSM community [58]. The study focused on peer-delivered interventions, with peers defined as “transgender men or transmasculine individuals.” Multiple investigators, all research staff interfacing with participants, and all intervention facilitators were transmasculine. A virtual community advisory board (CAB), composed of TMSM, was convened at study inception and met monthly or bimonthly via Zoom (Zoom Communications, Inc). CAB members received an honorarium for their time. The CAB played a central role in the research, collaborating closely with the study team on all aspects of the trial and serving as an essential mechanism for community consultation. The CAB provided extensive input on the interventions, study protocols and procedures, privacy and confidentiality safeguards, implementation and evaluation processes, recruitment and retention activities, dissemination strategies, and troubleshooting challenges to ensure acceptability for TMSM.

### Recruitment

Participants were recruited and enrolled virtually nationwide from US EHE jurisdictions [5]. Recruitment strategies included social media (eg, closed Facebook groups, Reddit), community networking platforms (eg, Lex), paid advertisements and posts on geosocial networking dating apps (eg, Grindr), outreach through transgender health care, social, and advocacy organizations (eg, listservs, events), and peer-to-peer networks (eg, referrals from research staff, the CAB, and enrolled participants). Recruitment materials (eg, flyers, text, images, advertisements, taglines) were designed to support purposive sampling, such as geotargeting and incorporating diverse images of BIPOC and Latine MSM. Flyers and web-based advertisements directed potential participants to the study web page, where they could complete a screening form or contact the research team directly via email.

### Retention

For retention, the study team collected and regularly updated participants’ contact information (eg, email, phone, mailing address, and Facebook or Instagram handles), along with the contact details of 1-3 individuals who could help locate them if needed. Participants were contacted through their preferred communication method to receive study visit reminders. Enrollment and retention were tracked in REDCap (Research Electronic Data Capture; Vanderbilt University), a secure, web-based, Health Insurance Portability and Accountability Act (HIPAA)–compliant system.

### Study Procedures

Potential participants first completed a digital screener to assess preliminary eligibility. Those deemed eligible were scheduled for a virtual enrollment visit via Zoom with study staff. After eligibility was confirmed, participants completed a written informed consent process. Individuals who consented were conditionally enrolled and then completed a baseline survey and a DBS home collection kit for HIV and PrEP testing (described below). Participants who confirmed HIV negativity were fully enrolled.

After participants received eligible HIV test results, they were randomized, and study staff informed them of their assigned condition and scheduled any intervention sessions. Following communication of HIV results and randomization, all participants received an email with a password-protected link to the SOC materials housed on the study website.

### Assessments and Survey Measures

**Figure 2:**
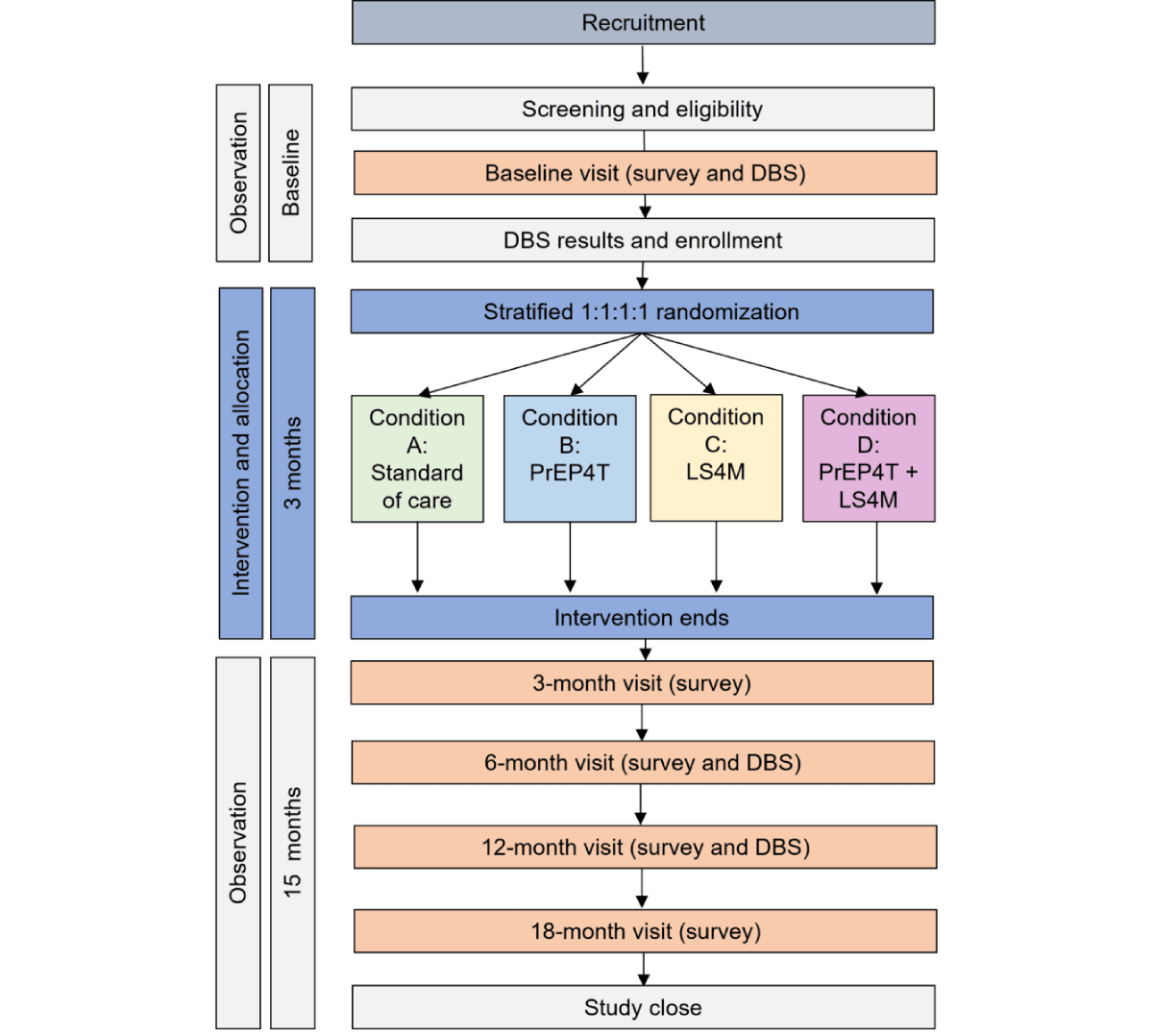
Study schema and flow diagram. DBS: dried blood spot; LS4M: LifeSkills for Men; PrEP4T: PrEP for transgender men and transmasculine people.

Participants completed online surveys at baseline and at 3, 6, 9, 12, 15, and 18 months (Figure 2). Comprehensive surveys, lasting approximately 30 minutes, were administered at baseline, 3, 6, 12, and 18 months. The 6-month survey also included an assessment of the intervention program completed after intervention delivery. Short surveys, focused on PrEP uptake and adherence and lasting about 15 minutes, were administered at 9 and 15 months. All surveys were hosted on REDCap, a HIPAA-compliant platform.

Survey measures were guided by the Healthcare Accessibility [49], Gender Affirmation [7,51], and IMB [53] frameworks. Validated measures from transgender populations and prior research were selected to ensure comparability [59-62]. Baseline surveys included both lifetime and past 3-month recall, while follow-up surveys assessed past 3-month recall only. Domains assessed included demographics (age, race, ethnicity, gender identity, sexual orientation, education, income, housing, health insurance), gender affirmation (social, medical, legal), and unmet gender affirmation needs [59,61]. Measures included the PrEP cascade (awareness, indications, uptake, adherence, and persistence, including both daily oral and long-acting injectable formulations) [15,62]; barriers and facilitators to PrEP care [8,62]; PrEP knowledge, motivations, and preferences [8,15,63]; PrEP self-efficacy [64]; and PrEP empowerment [65]. Behavioral risk for HIV acquisition was assessed through sexual partners and practices using an adaptation of the Transmasculine Sexual Health Assessment [66], as well as injection drug use, needle-sharing behaviors, and perceived HIV risk [62]. Sexual partner communication and affirmation were measured using the Sexual Communication Self-Efficacy Scale [67] and the Gender Non-Affirmation from Sexual Partners Scale [14], respectively. Additional questions addressed HIV testing outside the study and sexually transmitted infection screening and diagnoses [61]. Mental health measures included psychological distress (Kessler-6) [68], posttraumatic stress disorder symptoms (Primary Care Posttraumatic Stress Disorder Screen) [69], resilience (Brief Resilience Scale) [70], and use of mental health services (eg, psychotherapy, support groups). Alcohol use disorder was assessed with the Alcohol Use Disorders Identification Test—Consumption [71], substance use disorder symptoms with the Drug Use Disorder Identification Test—Consumption [72], along with categories of drugs used. Discrimination was measured using the Everyday Discrimination Scale [73], which included 14 possible attributions (eg, age, gender identity, race, ethnicity). Internalized stigma [61] was also assessed. Additional measures included health care empowerment [74], health care access, utilization, and mistreatment in health care settings [60]. Social support was assessed with a short form of the Social Support Questionnaire [75], the Trans Community Connectedness subscale of the Gender Minority Stress and Resilience Measure [76], and metrics of social network size [61].

### HIV Testing, Biomarker-Confirmed PrEP Outcomes, and Specimen Collection Protocol

The primary outcome was biomarker-confirmed PrEP uptake, assessed via emtricitabine triphosphate (FTC-TP) and tenofovir diphosphate (TFV-DP) concentrations in DBS. FTC-TP/TFV-DP assays were used for TFV-containing PrEP regimens approved for this population. PrEP uptake was defined as the detection of any quantifiable drug concentration. Secondary outcomes were PrEP adherence, indicated by TFV-DP concentrations >700 fmol/punch (corresponding to ≥4 doses per week) [54], and PrEP persistence, defined as 2 consecutive DBS samples showing PrEP use. Thresholds were based on the emtricitabine/tenofovir disoproxil fumarate (F/TDF) regimen and adjusted as appropriate for participants using other TFV-containing PrEP formulations (eg, emtricitabine/tenofovir alafenamide, F/TAF).

Participants self-collected DBS samples for HIV testing and PrEP level assessment at baseline, 6, and 12 months. Biospecimen processing was conducted by Molecular Testing Lab (MTL), a CAP-accredited and Clinical Laboratory Improvement Amendments–certified laboratory specializing in at-home specimen collection and processing. Participants provided a mailing address, which was validated through the US Postal Service Address Information application programming interface, and home specimen collection kits were shipped via the US Postal Service. Each kit included detailed visual instructions for specimen collection and a prepaid return mailer. Video instructions were available on the study website to guide participants through specimen self-collection. Each kit included a finger stick device, alcohol wipe, adhesive bandage, a Whatman 5-spot DBS card for HIV testing, and a Whatman 2-spot DBS card for PrEP adherence testing. Participants collected their specimens and returned the cards to the laboratory using the self-addressed, stamped envelope provided in the kit.

MTL conducted a fourth-generation HIV-1 antigen/antibody ELISA to detect HIV p24 antigen, HIV-1 (groups M and O) antibodies, and HIV-2 antibodies in human blood (sensitivity 100% and specificity 100%) (Molecular Testing Labs, unpublished data, December 2020). Biologic measures of PrEP uptake and cumulative adherence were performed only for participants who self-reported recent PrEP use. HIV test results were typically available within 14 days via the secure MTL portal, reviewed by study staff, and then shared with participants. Positive HIV results were communicated by designated clinical staff, who also contacted participants with positive DBS results to facilitate confirmatory testing. Participants with negative confirmatory results remained eligible for enrollment. PrEP levels were not reported to participants.

### Study Conditions and Interventions

#### Overview

The 4 study conditions are displayed in Table 1.

**Table 1:** Study conditions and sample size for the 2×2 factorial trial (n=300).

Study condition	Digital PrEP^a^ locator and digital library	One-on-one individualized peer health navigation	Peer-delivered group-based behavioral intervention	Sample size, n
A: SOC^b^	Yes	No	No	75
B: SOC + PrEP4T^c^	Yes	Yes	No	75
C: SOC + LS4TM^d^	Yes	No	Yes	75
D: SOC + PrEP4T + LS4TM	Yes	Yes	Yes	75

^a^PrEP: preexposure prophylaxis.

^b^SOC: standard of care.

^c^PrEP4T: PrEP for transgender men and transmasculine people.

^d^LS4TM: LifeSkills for Transgender Men and Transmasculine People.

#### Condition A (SOC)

Participants received access to a digital PrEP locator and resource library. The SOC included linkage to the Centers for Disease Control and Prevention’s (CDC’s) HIV Prevention Services Locator, a national directory of HIV testing and PrEP services [8,15,77,78]. A digital library of HIV prevention, sexual health, and antistigma materials (in written and video formats) was also provided. Content was curated from multiple sources, including the CDC [79], Gate Trans Men & HIV Project, UCSF Center for Excellence in Trans Health, and the National LGBTQIA+ Health Education Center. Resources covered transgender community support (eg, support groups), sexual health (eg, contraception, DoxyPEP), HIV prevention (eg, PrEP financial assistance programs, directories of transgender-affirming PrEP providers), and other medical services (eg, gender-affirming care providers). Participants accessed the SOC resource guide on the TOGETHR website through a personal account log-in assigned after randomization. Study staff regularly updated the resources, which could be filtered by topic and location.

#### Condition B (SOC + PrEP4T)

This is an online 1-on-1 peer navigation. PrEP4T was an individualized, TMSM-specific intervention adapted from The Fenway Model [41], consisting of online 1-on-1 sessions between a peer navigator and a participant using a strengths-based case management manualized curriculum (Tables 2 and 3) [80,81]. Peer navigators, employed by Fenway Health and sharing transmasculine community membership with participants, were trained to provide linkages and support. Using an individualized approach, they worked to identify and address barriers, apply an asset-based frame to promote resiliencies, and pragmatically leverage these to improve biomedical HIV prevention outcomes. PrEP4T included 9 hours of content delivered across six 1.5-hour weekly sessions, with peer navigators drawing on techniques from established behavior change models (eg, Motivational Interviewing [82,83]; Transtheoretical Model [84-86]) to support PrEP-related decision-making.

#### Condition C (SOC + LS4TM)

This is an online peer-delivered small group–based behavioral intervention. LS4TM was a theory-based, manualized intervention designed to reduce HIV risk and address mental health–related effects of stigma for TMSM [20]. The program consisted of 9 hours of content delivered through six 2-hour small group sessions (up to 12 participants per group) held weekly for 6 weeks (Tables 2 and 3). While the total content duration was the same as PrEP4T, additional time was allotted for extended group discussions. Sessions were facilitated by staff and contractors employed by Fenway Health, all of whom shared transmasculine community membership with participants.

#### Condition D (SOC + PrEP4T + LS4TM)

This is an online peer-delivered combined intervention. Participants in this arm received both PrEP4*t* (1-on-1) and LS4TM (group-based) interventions in addition to SOC. Group D received a total of 21 hours of content, consisting of six 2-hour LS4TM sessions and six 1.5-hour PrEP4T sessions. These interventions were not delivered simultaneously within the same 6-week period.

**Table 2:** PrEP4T^a^ intervention sessions: session number, title, content, and conceptual framework guiding the intervention sessions.

Session number	Session title	Session content	Conceptual framework
1	Introduction, Background, and Goal Setting	Build rapport with the participant Assess the participant’s background and sexual health history. Set a realistic, sexual health–related goal for the program (eg, to start PrEP^b^, to consistently communicate with partners about sexually transmitted infection testing). Discuss factors that may impact risk for HIV among transgender men and transmasculine people.	IMB^c^ and Gender Affirmation
2	HIV Transmission and Prevention	Assess what participants know about HIV prevention and how they currently put that information into practice. Introduce participants to the concept of harm reduction. Fill in knowledge gaps and answer questions about HIV prevention. Build on or reinforce participants’ existing harm reduction strategies.	IMB and Healthcare Accessibility
3	Accessing PrEP and Preventive Health Care	Discuss barriers to accessing PrEP and work with participants to identify solutions and options available to them. Provide a brief overview of where and how participants can access postexposure prophylaxis. Discuss preventive health care access, with an emphasis on primary care and sexually transmitted infection testing. Address other preventive care topics, including vaccinations (human papillomavirus, hepatitis A and B), contraception, cervical and anal Papanicolaou testing, and gender-affirming care.	IMB and Gender Affirmation Healthcare Accessibility
4	Partners and Communication	Define the key components of sexual health to help participants understand how supporting their physical, emotional, mental, and social well-being is part of sexual health. Discuss healthy relationships, partner communication, and boundary setting; share communication strategies and practice communication skills with scripting or role playing. Discuss self-care practices as a tool for managing stressors participants may encounter while communicating their sexual health needs to clinicians and partners, or while navigating the world as a “transgender man or transmasculine person” generally.	IMB and Gender Affirmation
5	Social Support	Identify resources and support systems that can support HIV prevention and sexual health goals. Use self-care practices and positive experiences of our transgender identities to build resilience.	IMB, Gender Affirmation, and Healthcare Accessibility
6	Program Summary and Wrap Up	Summarize program framing and discuss how social stress and IMB concepts connect with the ongoing HIV prevention work the participant has been engaged in. Identify long-term goals, as well as supports and resources participants can access after the conclusion of the program. Solicit verbal feedback, in addition to completion of the session and program feedback forms.	IMB

^a^PrEP4T: PrEP for transgender men and transmasculine people.

^b^PrEP: preexposure prophylaxis.

^c^IMB: Information, Motivation, and Behavioral Skills.

**Table 3:** LS4TM^a^ (small group–based intervention) intervention sessions: session number, title, content, and conceptual framework guiding the intervention sessions.

Session number	Session title	Session content	Conceptual framework
1	Identity Recognition and Affirmation	Provide an overview of the program. Discuss identities and reflect on the diversity of ways participants occupy different identities. Identify the misconceptions and stigmas that exist around the transgender men and transmasculine community, and how these have affected how people feel about their own identities. Discuss factors that may impact risk for HIV among transgender men and transmasculine people.	Gender Affirmation
2	Let’s Talk About Sex	Inform participants about the basics of HIV transmission, barriers, and safer injection practices.	IMB^b^
3	PrEP^c^ for Pleasure	Inform participants about PrEP, postexposure prophylaxis, and contraception. Encourage participants to consider whether PrEP may be right for them. Identify barriers to PrEP use and uptake, and ways to navigate these.	IMB and Healthcare Accessibility
4	Barriers and Opportunities	Learn about setting attainable goals and set a personal goal. Identify roadblocks and structural barriers that impact individual and collective goals and constrain decision-making. Discuss support and resources that can help to overcome roadblocks and barriers to achieving goals.	IMB and Healthcare Accessibility
5	Communication and Partner Negotiation	Identify our rights and responsibilities in relationships. Discuss a variety of partner communication topics relevant to transgender men and transmasculine people, including meeting partners, disclosing identities, educating partners about our transgender identities, and establishing consent and boundaries. Brainstorm and practice healthy partner communication and negotiation skills.	IMB and Gender Affirmation
6	Tying It All TOGETHR^d^	Highlight general sexual health concerns for transgender men and transmasculine people, including healthy sexuality and sexual pleasure. Review the program, then revisit and revise goals moving forward. Gather feedback from participants on the program, the curriculum, and how it was delivered.	IMB

^a^LS4TM: LifeSkills for Transgender Men and Transmasculine People.

^b^IMB: Information, Motivation, and Behavioral Skills.

^c^PrEP: preexposure prophylaxis.

^d^TOGETHR: Transmasculine One-on-One and Group Empowerment for Targeted HIV Reduction.

### Incentives and Disbursement

All participants received US $30 for each comprehensive survey completed (baseline, 3, 6, 12, and 18 months) and US $10 for each short survey completed (9 and 15 months). Participants received US $20 for each self-collected HIV/PrEP test kit completed at baseline, 6, and 12 months. Participants in the PrEP4T and LS4TM arms received US $10 for each intervention session attended (6 sessions per intervention). The total compensation for participants was as follows: condition A (SOC; US $250); condition B (PrEP4T; US $310); condition C (LS4TM; US $310); and condition D (PrEP4T + LS4TM; US $370). Participants completing a qualitative interview at 9 months received an additional US $50.

Participants were compensated using ClinCard, a reloadable debit card that allowed study staff to add funds via an online portal as study activities were completed. ClinCards were mailed directly to participants’ preferred addresses after study enrollment.

In the final 6 months of this 5-year study, PrEP4T and LS4TM were offered to all participants regardless of study group assignment to gather implementation data; no compensation was provided during this period.

### Qualitative Data Collection

A subset of 40 TMSM, stratified by PrEP use, was to be purposively selected for IDIs (10 per study arm) at their 9-month visit. Sampling aimed to ensure that 50% of participants were BIPOC or Latine. Additional participants could be recruited as needed to achieve data saturation. IDIs were conducted as part of the 9-month study assessment—the acute postintervention time point—to maximize recall. IDIs explored participant decision-making and experiences with PrEP and the interventions. Interviews were conducted via an online video-conferencing platform (Zoom) [87]. Each IDI lasted approximately 60-90 minutes, followed a semistructured interview guide (Multimedia Appendix 2), and was to be conducted by an interviewer trained in qualitative research and experienced in working with transgender communities.

### Randomization and Blinding

Participants were randomized after their baseline HIV results were received and confirmed. Randomization was automated, computer-generated, and allocation sequences were concealed. Stratification was based on ethnoracial identity (BIPOC or Latine vs White) and MAP state LGBTQ equality policy tally (“high, medium, or fair” vs “low or negative” overall policy tally, as of October 2023; see Table S2 in Multimedia Appendix 1) [55]. The MAP state LGBTQ equality policy tally assesses over 50 LGBTQ-related laws and policies across US states (eg, nondiscrimination, health care access, religious exemptions, identity documents), providing a snapshot of the legal and policy climate for LGBTQ people [55]. Stratification was implemented to ensure balance across study arms and to facilitate subgroup analyses [88], given the disproportionate HIV burden among BIPOC or Latine versus White individuals [5] and the impact of repressive anti-LGBTQ policies on HIV prevention efforts [89]. Blinding was maintained at the statistician and investigator levels [90], but was not possible for participants or intervention staff.

### Training and Quality Assurance/Quality Control

All research staff completed extensive training on the study objectives, protocol, and intervention procedures. Additional training covered transgender health, HIV, PrEP navigation, mental health crisis management, substance use, harm reduction, trauma-informed care, and addressing experiences of violence. Intervention staff training included observing and conducting mock intervention sessions. A mental health clinician, who was both a consultant and a member of the community, was available on-call to provide regular clinical supervision. Staff were also trained in human participant protections, confidentiality and privacy, informed consent, and Good Clinical Practice in accordance with federal regulations. Refresher training was conducted annually. Laboratory testing followed standardized protocols and procedures. Surveys were administered using computer-assisted self-interview methods to ensure consistency and reduce social desirability bias. Instruments were piloted to maximize comprehension, and logic checks and skip patterns were employed to minimize data entry errors. Data were reviewed regularly to ensure consistency and accuracy.

### Intervention Fidelity

To ensure interventions were implemented as intended and core components were delivered according to protocol, intervention facilitators completed a checklist documenting their self-rated adherence to the intervention procedures. Session notes captured qualitative data on intervention implementation, including facilitator rapport, group dynamics, and other insights related to delivering manualized interventions. At the 6-month follow-up survey, participants reported on the intervention content they received and their experiences, including responses on the 8-item Client Satisfaction Questionnaire [91].

### Statistical Analysis

#### Power

Factorial trials can be powered to detect either main effects or interaction effects, depending on the anticipated influence of interactions within the trial context [92]. This trial was powered to detect the main effects of the PrEP4T and LS4TM interventions. The trial was not powered to detect a PrEP4T × LS4TM interaction effect because (1) based on our conceptualization, the interventions were distinct in delivery (individualized vs group based) and content (peer navigation vs. community support and IMB strategies), and informed by preliminary studies and knowledge of the study population, we did not anticipate a combined interaction effect; and (2) any potential interaction was expected to be negligible, described in factorial trials as a minor “small quantitative interaction” [92-94]. Both PrEP4T and LS4TM were distinct, peer-delivered digital interventions that TMSM endorsed for HIV prevention in our prior research. We hypothesized that each strategy would independently increase PrEP uptake by at least 20% among TMSM, addressing the main-effects research question. Although we did not anticipate interaction effects, we hypothesized additive effects such that participants assigned to the PrEP4T + LS4TM condition would achieve the highest PrEP uptake rates. These superiority hypotheses were supported by evidence from a systematic review of peer-delivered interventions [47].

We estimated PrEP uptake rates in the SOC condition based on prior TMSM data [8,15] and conducted simulations using an SAS macro [95]. Simulations varied the number of participants per condition (n=40-120) and the magnitude of intervention effects over 18 months (20%-40%) for the PrEP4T and LS4TM conditions. Assuming 10% attrition, a total sample size of 300 (75 per condition) provided 80% power or greater to detect a 20% increase in PrEP uptake for each intervention versus SOC at a .05 significance level. We selected a 20% increase because smaller differences were not considered clinically meaningful for potential scale-up. To account for attrition, we planned to enroll 375 participants at baseline, anticipating approximately 20% loss to follow-up, resulting in 300 participants completing the study.

#### Data Analysis by Aim

We describe the planned analyses by aim.

Before analyses, data will be audited for quality and completeness. Descriptive statistics will be calculated, and variable distributions will be evaluated to ensure they meet the assumptions of planned analytic models, including detection of outliers. In this study, *t* tests and chi-square analyses will be used to assess group equivalence at baseline. Variables showing differences across the 4 groups will be considered for inclusion as covariates in the analyses. All analyses will be conducted using SAS (SAS Institute) or R (R Foundation) software, with statistical significance set at an α level of .05.

Aim 1 analyses will follow an intention-to-treat approach. Random-effects logistic regression will be used with PrEP uptake as the primary outcome. The model will include time (baseline, 6, 12, and 18 months), a categorical variable for study condition (PrEP4T, LS4TM, PrEP4T + LS4TM, with SOC as the reference), and the time × study condition interaction to test whether changes in PrEP uptake over time differ across the 3 intervention conditions compared with SOC. Baseline characteristics that differ across study conditions, as well as variables related to missingness, will be included as covariates if necessary. The primary parameter of interest is the time × study condition interaction term; a significant interaction would indicate that the rate of PrEP uptake over time differs between the intervention and SOC groups. Significant interactions will be graphed and interpreted. We hypothesize that PrEP uptake will be highest among participants randomized to condition D (PrEP4T + LS4TM). The same analytic approach will be applied to the secondary outcome of PrEP adherence. PrEP persistence will be assessed at months 12 and 18 using random-effects logistic regression, including time (12 and 18 months), study condition, and the time × study condition interaction. HIV and sexually transmitted infection incidence will be evaluated using logistic regression, with incidence between baseline and 18 months as the outcome and study condition as the predictor.

Aim 2 will use causal inference methods from epidemiology [96,97] to conduct mediation analyses and examine the mechanisms of action for the interventions. Mediation analyses will estimate the natural direct and indirect effects of proposed mediators—health care empowerment, TMSM community connectedness, and IMB constructs—on the relationship between intervention condition and PrEP uptake compared with SOC. Mediation models will be implemented using a series of random-effects logistic regression models, consistent with the approach described in aim 1.

For aims 1 and 2, patterns of missingness will be examined, and baseline responses will be compared between participants with and without missing data. Variables associated with missingness will be included in the analyses to yield valid inferences [98]. Modern missing data techniques [99] will be employed, with multiple imputation used to handle missing data, including participants lost to follow-up [100]. Sensitivity analyses will compare parameter estimates from completer-only analyses with those from multiple imputation to assess potential bias.

Aim 3 qualitative data from IDIs will be analyzed to explore PrEP decision-making and participant experiences with the interventions. An iterative analytic process will follow Crabtree and Miller’s [101] 5-step approach: (1) describing, (2) organizing, (3) connecting, (4) corroborating, and (5) representing. Regular debriefing meetings with data collectors will identify preliminary findings and guide iterative data collection and analysis. An initial set of codes will be developed based on the study’s conceptual frameworks of Healthcare Access, Gender Affirmation, and IMB constructs. Qualitative codes will be applied to transcripts and interview notes using qualitative analysis software by 2 independent analysts. New codes will be added as themes emerge. Summaries will be created for major themes and differences across subgroups. Descriptive quantitative survey data (eg, demographics, social and medical gender affirmation) may be used to contextualize qualitative findings. CAB members will provide ongoing input and help corroborate the qualitative results.

#### Data Analysis for Protocol Manuscript

Descriptive statistics were calculated for cumulative enrollment numbers, recruitment activities, screener and baseline survey completions, and sociodemographic characteristics of enrolled participants. To evaluate the effectiveness of different recruitment strategies, Granger causality tests [102] were conducted to forecast screening and baseline survey completions based on recruitment activity types; *F* tests on lagged values were used to determine whether recruitment strategies statistically predicted future screening and enrollment completions at 1- and 2-week intervals following recruitment activities, with significance set at an α level of .05. Trends over time were visualized graphically.

### Ethical Considerations

The Fenway Institute at Fenway Health served as the single institutional review board of record (institutional review board protocol approval number 2023-12). Written informed consent was obtained from all participants before initiating any study procedures. Extensive safeguards were implemented to protect participant information and ensure privacy and confidentiality, including storing participant names and contact information separately in password-protected files from deidentified survey data and HIV testing results. The study was protected by a Certificate of Confidentiality from the NIH. Participants were compensated for their involvement (see the “Incentives and Disbursement” section). A Data Safety and Monitoring Board was established to oversee the trial’s progress, review safety data, and provide recommendations on whether the trial should continue based on its assessment of safety and research performance. The board convened at least annually.

## Results

### Community Advisory Board Collaboration in Study Start-Up Activities

#### Overview

Monthly or bimonthly meetings of the virtual CAB ensured close collaboration and active participation with the research team. All study protocols and procedures were codeveloped and refined with CAB input to maximize acceptability for the study population. The CAB provided invaluable guidance throughout the study.

#### Study Population Definitions

Discussions with the CAB focused on defining and operationalizing the study population of “TMSM” to maximize inclusivity while targeting those at risk for HIV. This included considerations of gender identity (transgender men, nonbinary people, transmasculine individuals, and other gender-diverse people), sexual orientation identity (gay, bisexual, queer, and other diverse identities), and behavioral HIV risk criteria (gender and anatomy of sexual partners).

#### Operationalization of Geographic Stratification

Geographic stratification for randomization was discussed, focusing on ensuring balance across EHE geographic hot spots and capturing the geo-sociopolitical climate, including policy contexts that could influence PrEP access and the lived experiences of TMSM. The decision to incorporate MAP legislative maps resulted from consultation with the CAB.

#### Refinement of Study Design

The initial study design included a 3-month run-in period intended to reduce study attrition and mitigate loss-to-follow-up among participants who might enroll solely to complete the baseline assessment and HIV testing for the incentive. However, the CAB recommended removing the run-in period, noting that the waiting period could discourage participation, create unnecessary anxiety while participants awaited the interventions, and erode trust with the community, given the historical mistrust in medical and research settings due to discrimination and mistreatment.

#### Branding and Detailed Recruitment Plan

A study logo, branding, website, and recruitment materials were developed to promote diverse participation across race, ethnicity, and geographic regions (see Tables S3 and S4 in Multimedia Appendix 1). Digital flyers and online advertisements were created in collaboration with a paid consultant who was a member of the community. A detailed, stepwise recruitment plan with targeted dates was established to implement different strategies, enabling the team to track screening and enrollment numbers and evaluate the effectiveness of each recruitment approach.

### Recruitment and Enrollment

**Figure 3:**
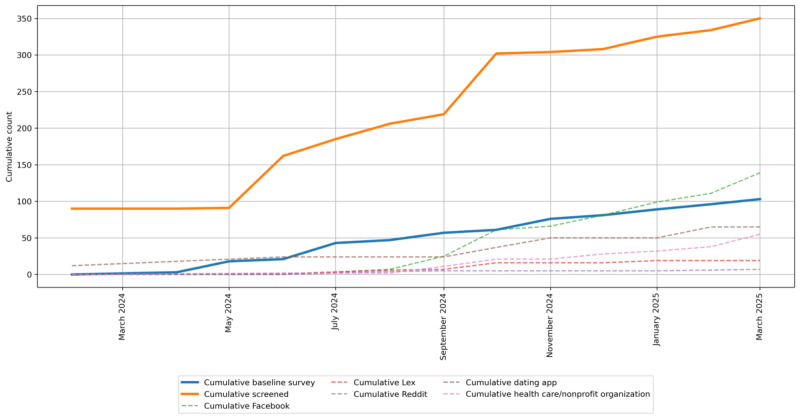
Cumulative recruitment and enrollment numbers by recruitment activity type (dashed lines), completed screeners (orange line), and completed baseline surveys (blue line), February 2024-March 2025.

Recruitment and enrollment began on February 1, 2024. Cumulative recruitment and enrollment numbers from February 2024 to March 2025 are shown in Figure 3. Recruitment activity types are represented by colored dashed lines, while completed screeners and completed baseline surveys are shown as solid orange and blue lines, respectively.

Granger causality tests were conducted to forecast screening and baseline enrollment survey completions based on recruitment activity types (Table 4). The most effective strategies for 2-week baseline survey completions were closed Facebook groups (*F*_2,23_=6.52, *P*=.006) and Lex (*F*_2,23_=4.63, *P*=.02). Lex also significantly predicted screening completions at both 1 week (*F*_1,26_=10.41, *P*=.003) and 2 weeks (*F*_2,23_=6.18, *P*=.007).

**Table 4:** Granger causality test forecasting the effect of recruitment efforts^a^ on screener and baseline enrollment survey completion with 1- and 2-week lag times, February 2024-March 2025.

Recruitment category	Survey type	Granger *F* test (*df*)^b^ for 1-week lag	*P* value^b^	Granger *F* test (*df*) for 2-week lag	*P* value^b^
Dating app	Screened	0.40 (1, 26)	.53	0.28 (2, 23)	.76
Dating app	Baseline survey	1.70 (1, 26)	.20	1.06 (2, 23)	.36
Facebook	Screened	0.58 (1, 26)	.45	0.43 (2, 23)	.65
Facebook	Baseline survey	0.07 (1, 26)	.80	6.52 (2, 23)	*.006*
Health care/nonprofit organization	Screened	1.44 (1, 26)	.24	1.16 (2, 23)	.33
Health care/nonprofit organization	Baseline survey	2.64 (1, 26)	.12	2.05 (2, 23)	.15
Lex	Screened	10.41 (1, 26)	*.003*	6.18 (2, 23)	*.007*
Lex	Baseline survey	1.17 (1, 26)	.29	4.63 (2, 23)	*.02*
Reddit	Screened	0.43 (1, 26)	.52	0.21 (2, 23)	.81
Reddit	Baseline survey	0.48 (1, 26)	.49	2.69 (2, 23)	.09

^a^Recruitment effort represents the contacting of an organization or posting of social media or dating app advertisements.

^b^Italicized *P* values indicate statistical significance at =.05.

As of March 20, 2025, 103 TMSM were enrolled (Table 5). The median age was 26 (IQR 24-31) years. Among the 103 participants, 39 (37.9%) identified as BIPOC and 18 (17.5%) identified as Latine. The most frequently reported gender identities were “transgender men or trans man” (36/103, 35%); transmasculine (31/103, 30.1%); and nonbinary, genderqueer, or gender nonconforming (15/103, 14.6%). The majority of participants had socially, legally, and medically affirmed their gender identity. Participants reported diverse sexual orientation identities, with 67 (65%) identifying as queer, 38 (36.9%) as bisexual, 37 (35.9%) as gay, and 18 (17.5%) as pansexual (totals exceed 100% due to a “select all that apply” response format). The sample was highly educated: 69 (67%) had a 4-year college degree or higher, and 24 (23.3%) were full-time students. A small proportion (7/103, 6.8%) reported having no health insurance.

**Table 5:** Sociodemographics of enrolled participants (N=103).

Sociodemographics		Values
Age in years, median (IQR)		26 (24-31)
**Categorical age (years), n (%)**		
	18-24	36 (35.0)
	25-29	33 (32.0)
	30-39	28 (27.2)
	40+	5 (4.9)
	Missing	1 (1.0)
**Gender identity (single option), n (%)**		
	Man	13 (12.6)
	Male	1 (1.0)
	Man of transgender experience	2 (1.9)
	“Transgender man or trans man”	36 (35.0)
	Female-to-male	3 (2.9)
	Transmasculine	31 (30.1)
	Nonbinary	7 (6.8)
	Genderqueer	6 (5.8)
	Gender nonconforming	2 (1.9)
	Agender	2 (1.9)
**Sexual orientation (select all-that-apply), n (%)**		
	Straight or heterosexual	1 (1.0)
	Gay	37 (35.9)
	Bisexual	38 (36.9)
	Queer	67 (65.0)
	Pansexual	18 (17.5)
	Asexual	2 (1.9)
	Demisexual	10 (9.7)
	Questioning or unsure	2 (1.9)
	I do not label my sexual orientation	9 (8.7)
	Another sexual orientation	5 (4.9)
**Racial identity, n (%)**		
	White	64 (62.1)
	Asian	5 (4.9)
	Black or African American	5 (4.9)
	Latino/e or Hispanic	8 (7.8)
	Multiracial	21 (20.4)
**Latino/ethnicity, n (%)**		
	Yes	18 (17.5)
	No	85 (82.5)
**Health insurance type^a^, n (%)**		
	No health insurance	7 (6.8)
	Public health insurance	17 (16.5)
	Private health insurance	79 (76.7)
**US Census region, n (%)**		
	Midwest	21 (20.4)
	Northeast	30 (29.1)
	South	30 (29.1)
	West	19 (18.4)
	Ending the HIV Epidemic Area, not specified	3 (2.9)
**Highest educational attainment, n (%)**		
	High school diploma or General Educational Development	6 (5.8)
	Associate’s degree/trade school certification	3 (2.9)
	Some college or university	25 (24.3)
	Bachelor’s degree	43 (41.7)
	Some graduate school	10 (9.7)
	Graduate degree	16 (15.5)
**Current student status, n (%)**		
	No	72 (69.9)
	Yes, part time	7 (6.8)
	Yes, full time	24 (23.3)
**Outwardly express gender identity full time, n (%)**		
	Yes	91 (88.3)
	No	12 (11.7)
**Any legal gender affirmation^b^, n (%)**		
	Yes	79 (76.7)
	No	24 (23.3)
**Gender-affirming hormone use (testosterone), n (%)**		
	Yes, within the last 3 months	94 (91.3)
	Yes, but not in the last 3 months	8 (7.8)
	Missing	1 (1.0)
**Any history of gender-affirming surgery, n (%)**		
	Yes	75 (72.8)
	No	28 (27.2)

^a^Participants could report more than 1 type of health insurance. If a participant reported both public and private insurance, they were coded as having private insurance. Additionally, participants who reported coverage through an employer, parents, partner, or college/university were also coded as having private insurance.

^b^Any legal gender affirmation was operationalized as having at least one identification document that listed the participant’s affirmed name or gender.

## Discussion

### Anticipated Findings

This digital factorial trial aimed to compare the efficacy of online 1-on-1 peer navigation (PrEP4T), online peer-delivered small group–based behavioral intervention (LS4TM), a combination of 1-on-1 and group-based interventions (PrEP4T + LS4TM), and an SOC in increasing PrEP uptake and adherence among TMSM in HIV epidemic priority hot spots nationwide. It represents the first full-scale efficacy trial of peer-delivered interventions with TMSM. The study also sought to enhance understanding of the pathways linking health care access, gender affirmation needs, community connectedness, and PrEP uptake and adherence in TMSM, with potential applicability to other transgender populations. This study was part of a critical research pathway aimed at identifying strategies to prevent HIV acquisition in TMSM, a population historically underserved in HIV prevention. Key lessons include the essential role of community participation through the CAB and the effectiveness of social media and community networking platforms for recruiting and engaging this population. Findings from this study can inform future trials of HIV prevention packages in which peer-delivered interventions are a core component.

### Limitations

Several limitations of this study should be noted. First, although the study was conducted nationwide, participants were drawn from EHE jurisdictions, limiting generalizability to the broader US population. Second, as a fully digital trial, participants were required to have access to a smartphone or computer with internet connectivity. This may have led to undercoverage of the source population and the unintentional exclusion of individuals with limited resources, restricted digital access, or intermittent technology access—groups who may also be at risk for HIV acquisition. Finally, this study cannot fully disentangle the effects of the intervention content from the additional time spent with peer navigators in the combined group. Nevertheless, the trial addresses a critical research gap by comparing individual versus group formats and evaluating the combined effects of both approaches within an at-risk and underresearched HIV epidemic population [47,48].

### Conclusions

This novel study aimed to address an urgent gap in HIV prevention research for an underserved and priority population at risk in the HIV epidemic. Close collaboration and partnership with the TMSM community, along with extensive community engagement activities, were central to the study’s success and provided valuable lessons for future HIV prevention research and practice with this population. Findings may also inform digital, peer-based HIV prevention strategies for other vulnerable populations affected by the HIV epidemic.

## Appendix

Multimedia Appendix 1 Additional analysis.

Multimedia Appendix 2 Interview guide.

## Abbreviations

BIPOC: Black, Indigenous, and People of Color
CAB: Community Advisory Board
CDC: Centers for Disease Control and Prevention
cis: cisgender
DBS: dried blood spot
EHE: Ending the HIV Epidemic
F/TAF: emtricitabine/tenofovir alafenamide
F/TDF: emtricitabine/tenofovir disoproxil fumarate
FTC-TP: emtricitabine triphosphate
HIPAA: Health Insurance Portability and Accountability Act
IDI: in-depth interview
IMB: Information, Motivation, and Behavioral Skills
LGBTQ: lesbian, gay, bisexual, transgender, and queer
LS4M: LifeSkills for Men
LS4TM: LifeSkills for Transgender Men and Transmasculine People
MAP: Movement Advancement Project
MSM: men who have sex with men
MTL: Molecular Testing Lab
NIH: National Institutes of Health
PrEP: preexposure prophylaxis
PrEP4T: PrEP for transgender men and transmasculine people
REDCap: Research Electronic Data Capture
SOC: standard of care
SPIRIT: Standard Protocol Items: Recommendations for Interventional Trials
TFV-DP: tenofovir diphosphate
TMSM: transgender men and transmasculine people who have sex with men
TOGETHR: Transmasculine One-on-One and Group Empowerment for Targeted HIV Reduction

## Data Availability

Deidentified individual-level data and a corresponding data dictionary will be made available upon reasonable request, following approval of a proposal and execution of a data use agreement. External requests to access TOGETHR data undergo review and approval by the principal investigator, coinvestigators, and the Community Advisory Board. Further details and request forms can be obtained by contacting SLR via email.
