# Supplementary material for: Transgender Men and Transmasculine One-on-One and Group-Delivered Empowerment for Targeted HIV Reduction (TOGETHR) Study: Protocol for a Digital Factorial Randomized Controlled Trial
Source: JMIR Res Protoc. 2025 Oct 20;14:e76831. doi: 10.2196/76831 (PMC12583947; doi:10.2196/76831)
Supplement: Multimedia Appendix 1 [file resprot_v14i1e76831_app1.docx]

**Multimedia Appendix 1**

**Table S1.** Ending the HIV Epidemic (EHE): Targeted Geographic Hotspots in the US for Study Enrollment

| **(A) Map of Prioritized Geographic Hotspots in the Ending the HIV Epidemic Strategy**  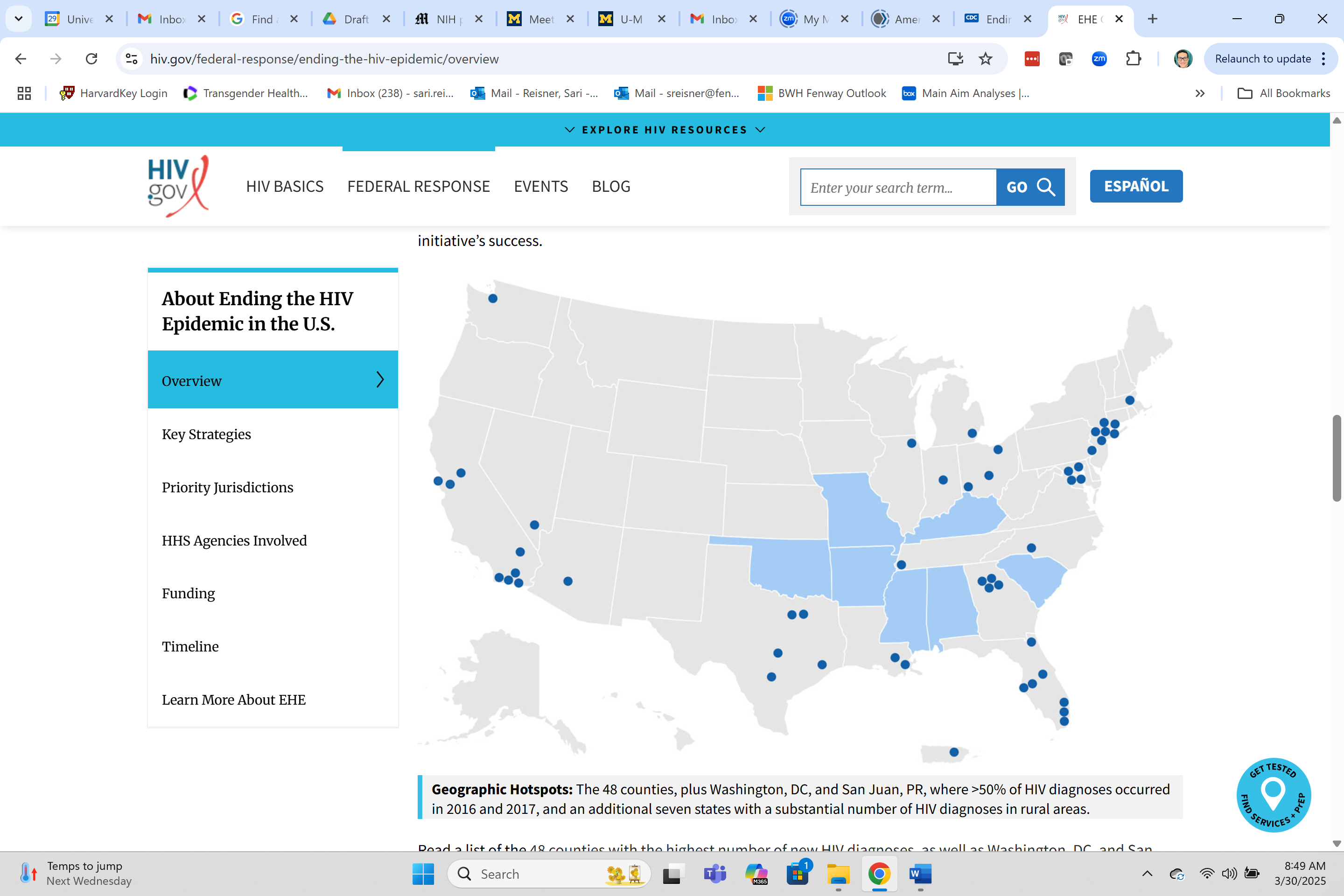  Geographic hotspots: 48 counties, plus Washington DC, and San Juan, Puerto Rico, where >50% of HIV diagnoses occurred in 2016 and 2017, and an additional 7 states with a substantial number of HIV diagnoses in rural areas. |
| --- |
| **(B) List of EHE-Targeted Counties and States**  **Counties:**   - Arizona   - Maricopa County - California   - Alameda County   - Los Angeles County   - Orange County   - Riverside County   - Sacramento County   - San Bernadino County   - San Diego County   - San Francisco County - Florida   - Broward County   - Duval County   - Hillsborough County   - Miami-Dade County   - Orange County   - Palm Beach County   - Pinellas County - Georgia   - Cobb County   - DeKalb County   - Fulton County   - Gwinnett County - Illinois   - Cook County - Indiana   - Marion County - Louisiana   - East Baton Rouge Parish   - Orleans Parish - Maryland   - Baltimore City   - Montgomery County   - Prince George’s County - Massachusetts   - Suffolk County - Michigan   - Wayne County - Nevada   - Clark County - New Jersey   - Essex County   - Hudson County - New York   - Bronx County   - Kings County   - New York County   - Queens County - North Carolina   - Mecklenburg County - Ohio   - Cuyahoga County   - Franklin County   - Hamilton County - Pennsylvania   - Philadelphia County - Tennessee   - Shelby County - Texas   - Bexar County   - Dallas County   - Harris County   - Tarrant County   - Travis County - Washington   - King County - Washington, D.C. - Puerto Rico   - San Juan Municipio   **States:**   - Alabama - Arkansas - Kentucky - Mississippi - Missouri - Oklahoma - South Carolina |

Source: Office of Infectious Disease and HIV/AIDS Policy, US Department of Health and Human Services (2019). “EHE Priority Jurisdiction.” <https://www.hiv.gov/federal-response/ending-the-hiv-epidemic/jurisdictions> (Accessed April 15, 2025).

**Table S1.** Movement Advancement Project (MAP) State LGBTQ Equality Policy Tally: Operationalization of Stratification for Randomization

| **(A) Movement Advancement Project (MAP) State LGBTQ Equality Policy Tally Map**  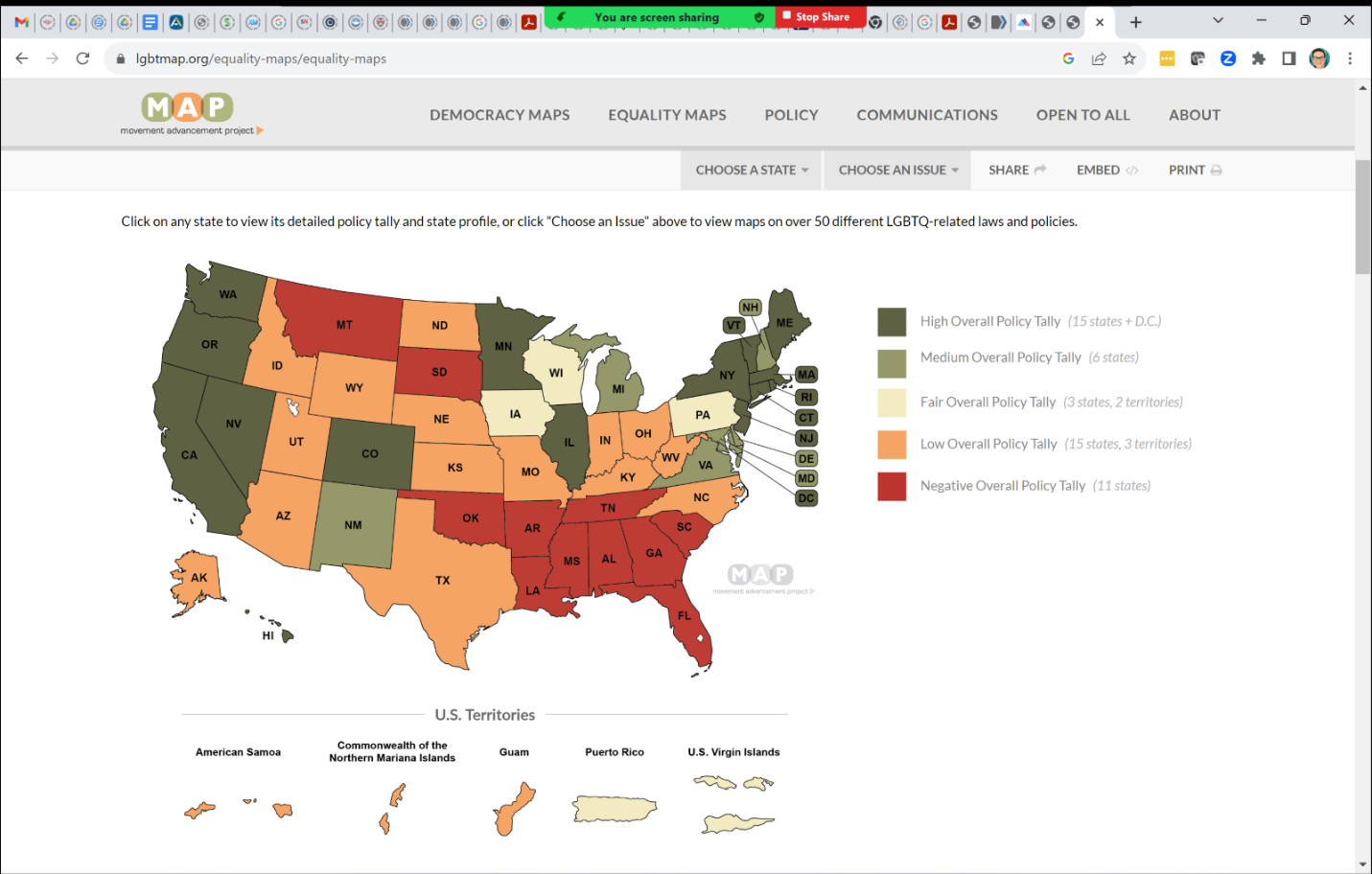  Participants are randomized 1:1:1:1 to each of the 4 study conditions stratified by race/ethnicity (Black, Indigenous, and People of Color [BIPOC] or White non-Latine) and Movement Advancement Project (MAP) state LGBTQ equality policy tally (“high, medium, or fair” overall policy tally or “low or negative” overall policy tally as of October 2023). |
| --- |
| **(B) Stratified Randomization**  Above MAP threshold (high, medium, or fair overall policy tally):   - Pennsylvania - Puerto Rico - California - Illinois - Maryland - Massachusetts - Nevada - New Jersey - New York - Washington - Washington D.C. - Michigan   Below MAP threshold (low or negative overall policy tally):   - North Carolina - Ohio - Arizona - Florida - Georgia - Indiana - Louisiana - Tennessee - Texas - Alabama - Arkansas - Kentucky - Mississippi - Missouri - Oklahoma - South Carolina |

Source: Movement Advancement Project (2023). "Equality Maps Snapshot: LGBTQ Equality by State." <https://www.mapresearch.org/equality-maps/> (Accessed October 17, 2023).

**Table S4.** Study Flyers and Advertisements

| **(A) Examples of Study Flyers**  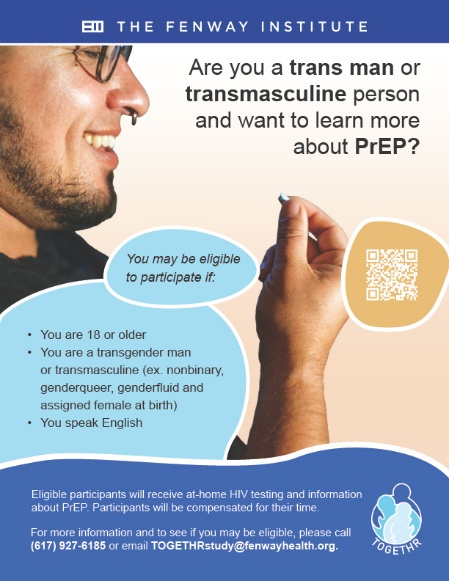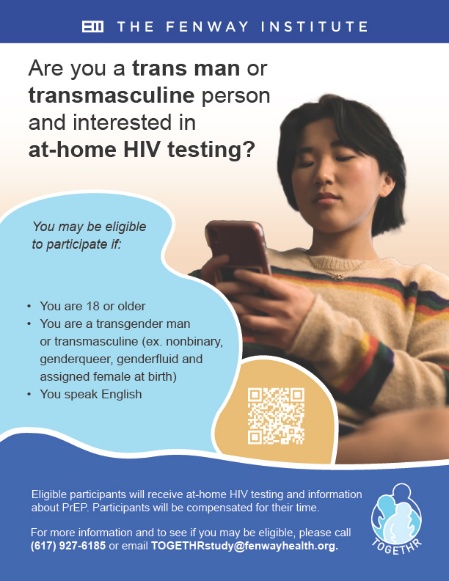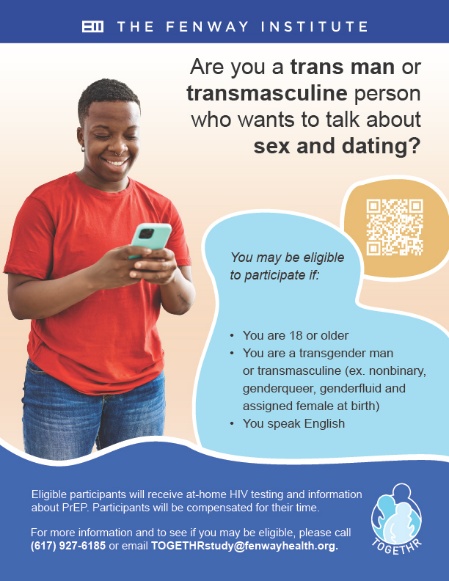 |
| --- |
| **(B) Examples of Study Online Advertisements**  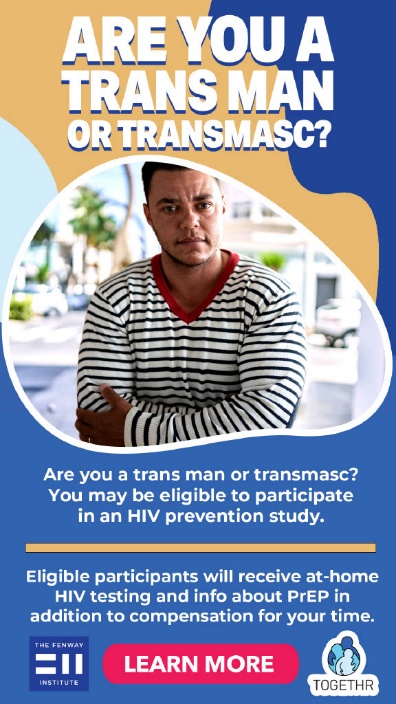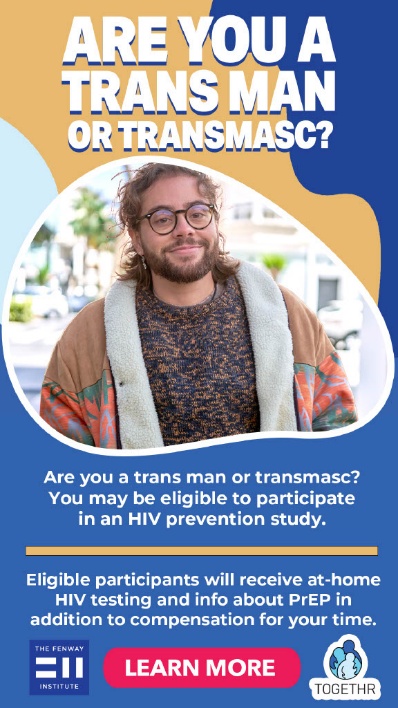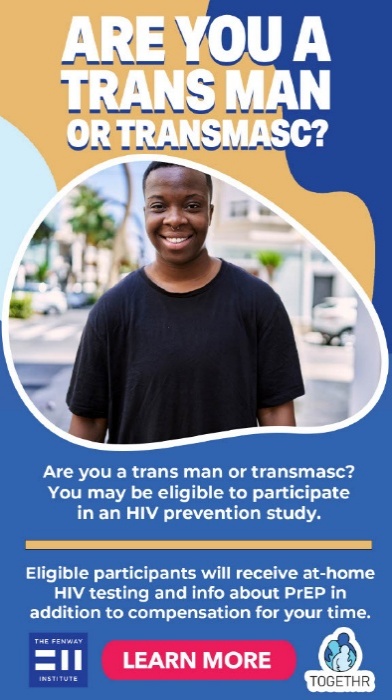 |

**Table S4.** Study Website and Website Resources

| **(A) Study Website Homepage**: <https://www.togethrstudy.org/>  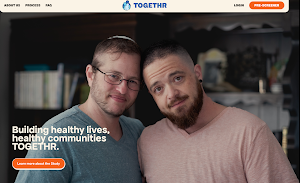  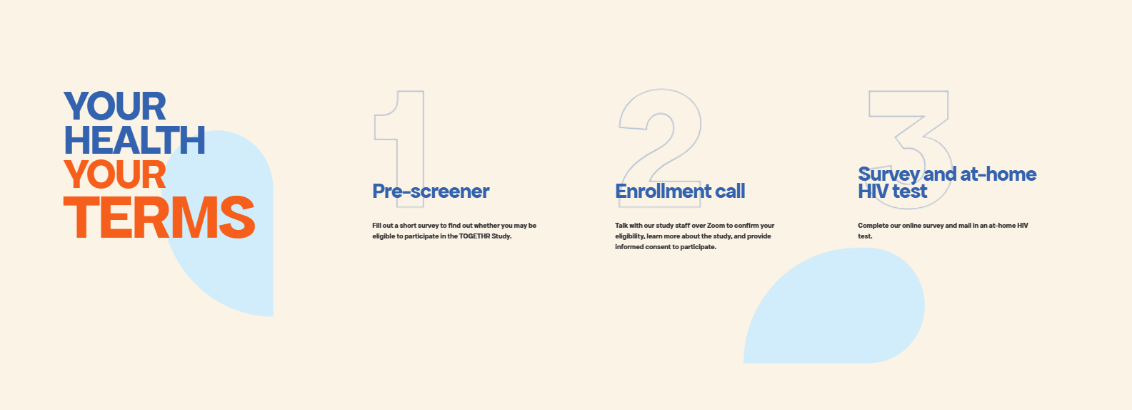 |
| --- |
| **(B) Study Website Resource Page: Searchable by Geographic Location and Topic**  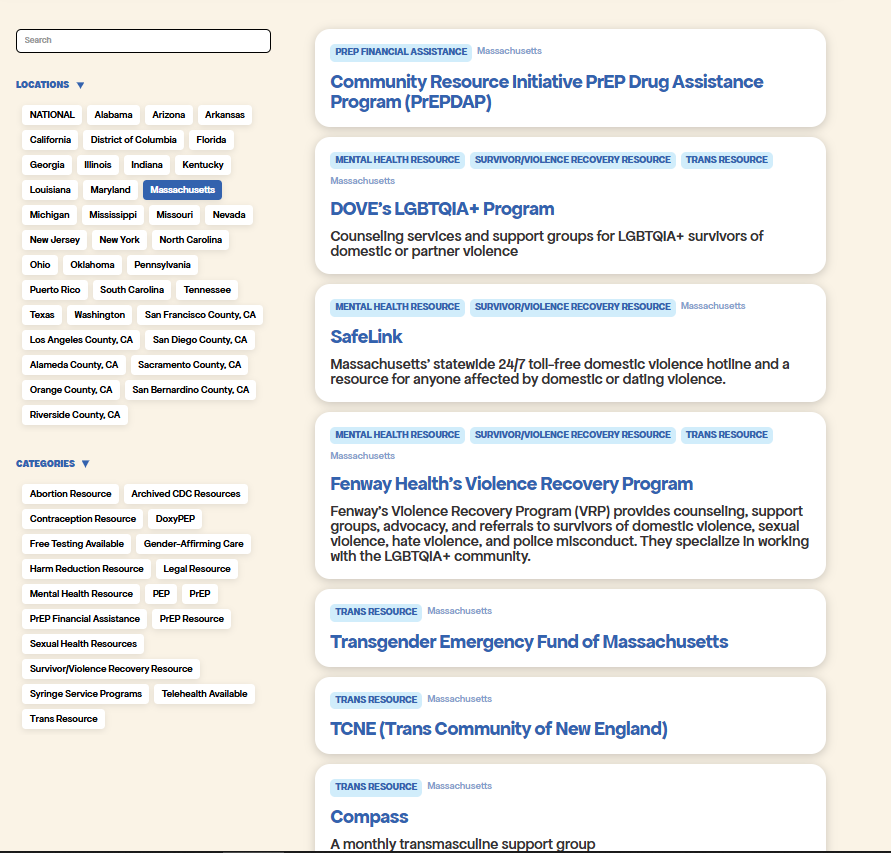 |
