# Supplementary material for: Transgender Men and Transmasculine One-on-One and Group-Delivered Empowerment for Targeted HIV Reduction (TOGETHR) Study: Protocol for a Digital Factorial Randomized Controlled Trial
Source: JMIR Res Protoc. 2025 Oct 20;14:e76831. doi: 10.2196/76831 (PMC12583947; doi:10.2196/76831)
Supplement: Multimedia Appendix 2 [file resprot_v14i1e76831_app2.docx]

TOGETHR Interview Guide_v1.1

Participant ID:

Interviewer:

Date:

Circle intervention:

1. LS4TM
2. PrEP4T
3. LS4TM + PrEP4T
4. SOC

Section designated as “**LS4TM**” are **only** for participants that are in the LS4TM or LS4TM + PrEP4T intervention

Section designated as “**PrEP4T**” are **only** for participants that are in the PrEP4T or LS4TM + PrEP4T intervention

Section designated as “**SOC**” are **only** for participants that are in the SOC intervention

Circle category:

1. Participant who initiated PrEP
2. Participant who did not initiate PrEP

Questions designated as “**Category A**” are **only** for participants that are in the category *Initiated PrEP*

Questions designated as “**Category** **B**” are **only** for participants that are in the category *Did Not Initiate PrEP*

**At the start of the discussion:**

Hello, my name is [name] and I use [pronouns]. Thank you for taking the time to do an interview today. Your thoughts and opinions are valuable, and I appreciate your willingness to help us understand people’s experiences with PrEP, how people make decisions to use or not use PrEP, and the experiences of people who participated in this study. I’ll be taking notes on what you say, and I’d also like to record the interview to make sure that I don’t miss anything you say. Your name will not be recorded. The recording will not be shared with anyone outside of the study team and will be destroyed after the study is complete. Taking part in this interview is completely voluntary and you are free to stop at any time. I encourage you to speak openly and honestly about your thoughts and experiences. Please keep in mind that there are no right or wrong answers to these questions. Some of these questions may be personal, so remember that you may skip questions, take breaks, or stop the interview for any reason, at any time. This interview will take about an hour. Does this all sound okay to you? Great, I am looking forward to learning more about these experiences from you. Do you have any questions for me before we begin?

**Introduction:**

1. First, I would like to start by getting to know you. Tell me a little bit about yourself. What does a typical day look like for you? [2-5 minutes]
   1. What do you like to do for fun?
   2. Tell me about your housing situation?
   3. How about friends, family, relationships, pets?
   4. *If applicable*: What do you do for school/work?
2. Next, I would like to learn about your experience of gender. How do you describe your gender? What are some ways that you have affirmed your gender? [~5 minutes]
   1. How do you describe your gender identity?
   2. What are some ways that you connect to the trans community or other trans people?
   3. What are some ways you’ve socially, medically, or legally affirmed or expressed your gender?
      1. *If they need specific prompting*: Some examples of how people may express or affirm their gender are changing their names, pronouns, ID documents, or how they dress; taking hormones; or having surgery.
3. I would also like to learn about your experience of sexuality. How do you describe your sexual orientation? [2-5 minutes]
   1. Has your sexual orientation evolved alongside your gender identity? If so, how?

**Motivation to participate:**

1. Why did you decide to participate in this research study?  [2-5 minutes]
   1. Have you taken part in other research studies in the past?
      1. *If yes:* What kind of study was it/what kinds of studies were they?
   2. How did you find out about this study?

1. What do you see as the benefits of participating in this study? [2-5 minutes]
   1. How did getting information about PrEP through the study affect your decision to participate in the study?
      1. Did you access PrEP navigation or counseling before participating in the study?
         1. *If yes:* How did you get access to PrEP navigation or counseling?
   2. How did getting home HIV testing through the study affect your decision to participate in the study?
      1. Did you get HIV or STI testing before participating in the study?
         1. *If yes:* How did you get access to HIV or STI testing?

**HIV and sexually transmitted infection (STI) risk perception:**

1. How much do you typically worry about exposure to HIV or STIs? [~10 minutes]
   1. What worries or concerns do you have about exposure to HIV or STIs?
      1. *If they don’t have worries/concerns:* What keeps you from worrying about exposure to HIV or STIs?
   2. Tell me about any experiences you’ve had with STIs.
      1. *If they need specific prompting*: Have you ever been diagnosed with an STI?
         1. *If yes:* How did you find out and what did you do about it the first time you were diagnosed with an STI? How did you find out and what did you do about it the most recent time you were diagnosed with an STI?
   3. Tell me about any times you have dated or had a sexual partner who was HIV+.
      1. How/when did you learn they were HIV+? How did you react?
      2. How did that affect you? How did that affect your sex life?
   4. How do you feel your risk for HIV or STIs compares to other people?
      1. Why do you think that your risk is lower/higher/the same as other people?
2. Tell me some examples of things you did before participating in this study to lower your chances of getting HIV or other STIs. [~10 minutes]
   1. *If they need specific prompting*: Tell me about your experiences with condoms, lubricant, meeting or selecting sexual partners, communicating with sexual partners, choosing what types of sex to have, and other ways you have tried to lower your risk of getting HIV or STIs.
   2. Has that changed throughout your participation in the study? If so, how? What do you think has prompted those changes?

**PrEP decision making**

1. What did you know about PrEP before participating in this study? [2-5 minutes]
   1. How did you first learn about PrEP?
   2. Where did you hear or learn about PrEP before participating in this study?
2. *Category A:* In this study, participants learn more about PrEP. Can you tell me about your decision to take PrEP? [~10 minutes]
   1. Can you tell me about your reasons for taking PrEP? How did you make that decision?
   2. What have been some benefits of taking PrEP for you? What have you liked about it?
   3. What are some challenges or things you’ve disliked about being on PrEP?
   4. What type of PrEP do you take? Can you tell me about your reasons for choosing that type of PrEP? How did you make that decision?
   5. *If oral PrEP:* Have you had any difficulty taking PrEP every day? What are some things that have gotten in the way of that, or things that have made it easier to do?
   6. Have you chosen to disclose that you are on PrEP to others? How did they react?
   7. Can you tell me about any times you decided against taking PrEP, or stopped taking PrEP for any amount of time? What were your reasons?
3. *Category A:* In this study, participants get referrals to PrEP providers. Can you tell me about your experiences accessing PrEP? [~10 minutes]
   1. How were you able to get access to PrEP? How easy or difficult was that for you?
4. *Category B:* In this study, participants choose whether or not to take PrEP. Can you tell me about your decision to not take PrEP? [~10 minutes]
   1. Have you ever taken PrEP in the past?
      1. *If yes:* What were the reasons you chose to stop taking PrEP?
   2. What would make you consider taking PrEP?
      1. *Potential probes:* Change in partner? Change in how often or how you have sex? Being able to take PrEP a different way? Being able to access PrEP a different way?
   3. How do you think people in your life or members of your community would react to you or someone else taking PrEP?

**Intervention experiences: LS4TM**

1. Can you tell me about your experience in the peer group program? [~10 minutes]
   1. What did you enjoy most about the program? (*Probes:* peer support, connecting with other transmasculine people, specific activities, information received, referrals to services, access to sexual health or gender-affirming resources, help creating positive goals or behavior changes, individual or group facilitators.)
   2. What was especially useful or relevant about the program for you personally?
   3. In what ways did the program meet your needs?
   4. What was your least favorite part of the program?
   5. In what ways did the program not meet your needs?
   6. Would you be interested in participating in this type of program again? Why or why not?
   7. Would you recommend this program to others? Why or why not?
   8. What would you change about the program, if you could?
   9. What did you think of the digital format of the program, compared to a program delivered in-person or face-to-face?
   10. What do you think is needed to make this program a success in the future?
   11. What characteristics do you think are needed for a good peer facilitator in this program?
   12. Please share any other feedback you have about the program.

**Intervention experiences: PrEP4T**

1. Can you tell me about your experience in the one-on-one peer navigation program? [~10 minutes]
   1. What did you enjoy most about the program? (*Probes:* peer support, connecting with other transmasculine people, specific activities, information received, referrals to services, access to sexual health or gender-affirming resources, help creating positive goals or behavior changes, individual or group facilitators.)
   2. What was especially useful or relevant about the program for you personally?
   3. In what ways did the program meet your needs?
   4. What was your least favorite part of the program?
   5. In what ways did the program not meet your needs?
   6. Would you be interested in participating in this type of program again? Why or why not?
   7. Would you recommend this program to others? Why or why not?
   8. What would you change about the program, if you could?
   9. What did you think of the digital format of the program, compared to a program delivered in-person or face-to-face?
   10. What do you think is needed to make this program a success in the future?
   11. What characteristics do you think are needed for a good peer facilitator in this program?
   12. Please share any other feedback you have about the program.

**Intervention experiences: SOC**

1. Can you tell me about your experience receiving information about PrEP designed for trans men and transmascs? [~10 minutes]
   1. What did you enjoy most about the program? (*Probes:* peer support, connecting with other transmasculine people, specific activities, information received, referrals to services, access to sexual health or gender-affirming resources, help creating positive goals or behavior changes, individual or group facilitators.)
   2. What was especially useful or relevant about the program for you personally?
   3. In what ways did the program meet your needs?
   4. What was your least favorite part of the program?
   5. In what ways did the program not meet your needs?
   6. Would you be interested in participating in this type of program again? Why or why not?
   7. Would you recommend this program to others? Why or why not?
   8. What would you change about the program, if you could?
   9. What did you think of the digital format of the program, compared to a program delivered in-person or face-to-face?
   10. What do you think is needed to make this program a success in the future?
   11. What characteristics do you think are needed for a good peer facilitator in this program?
   12. Please share any other feedback you have about the program.

**Conclusion**

1. Do you have any additional experiences, feedback, ideas, or suggestions that you would like to share or discuss?

**Interview Summary**

| **Code or Demographic** | **Summary and/or Salient Quote** |
| --- | --- |
| Age |  |
| Hormone use |  |
| PrEP use |  |
| Study motivation |  |
| Risk perception |  |
| PrEP rationale |  |
| PrEP downsides |  |
| PrEP benefits |  |
| PrEP barriers |  |
| PrEP facilitators |  |
| Interactions |  |

**Complete the section below after the interview is over and the participant has left.**

Summary of participant background:

Summary of interaction with participant (their mood, comfort, rapport, etc.):

Reflexive Notes (how did the interviewer feel about the interview and the interaction with the participant):
